# LED Light-Induced ROS Differentially Regulates Focal Adhesion Kinase Activity in HaCaT Cell Viability

**DOI:** 10.3390/cimb44030082

**Published:** 2022-03-04

**Authors:** Jun-Sub Kim, Ssang-Taek Steve Lim

**Affiliations:** 1 Department of Biotechnology, Korea National University of Transportation, Jeungpyeong 27909, Chungbuk, Korea; 2 Department of Biochemistry and Molecular Biology, College of Medicine, University of South Alabama, Mobile, AL 36688, USA

**Keywords:** FAK, LED light, ROS, HaCaT, skin

## Abstract

In this study, changes in cell signaling mechanisms in skin cells induced by various wavelengths and intensities of light-emitting diodes (LED) were investigated, focusing on the activity of focal adhesion kinase (FAK) in particular. We examined the effect of LED irradiation on cell survival, the generation of intracellular reactive oxygen species (ROS), and the activity of various cell-signaling proteins. Red LED light increased cell viability at all intensities, whereas strong green and blue LED light reduced cell viability, and this effect was reversed by NAC or DPI treatment. Red LED light caused an increase in ROS formation according to the increase in the intensity of the LED light, and green and blue LED lights led to sharp increases in ROS formation. In the initial reaction to LEDs, red LED light only increased the phosphorylation of FAK and extracellular-signal regulated protein kinase (ERK), whereas green and blue LED lights increased the phosphorylation of inhibitory-κB Kinase α (IKKα), c-jun N-terminal kinase (JNK), and p38. The phosphorylation of these intracellular proteins was reduced via FAK inhibitor, NAC, and DPI treatments. Even after 24 h of LED irradiation, the activity of FAK and ERK appeared in cells treated with red LED light but did not appear in cells treated with green and blue LED lights. Furthermore, the activity of caspase-3 was confirmed along with cell detachment. Therefore, our results suggest that red LED light induced mitogenic effects via low levels of ROS–FAK–ERK, while green and blue LED lights induced cytotoxic effects via cellular stress and apoptosis signaling resulting from high levels of ROS.

## 1. Introduction

The scientific basis of photodynamic therapy (PDT) was defined at the beginning of the twentieth century [1], and currently, the main dermatological application of PDT is as a low-level laser therapy [2]. Recently, light-emitting diode (LED) irradiation has been the focus of research concerning PDT [2], as LEDs can be an effective and alternative light source, providing advantages due to their broad beam width and cost efficiency. In addition, evidence has increasingly suggested beneficial effects of LEDs in the treatment of many conditions such as skin inflammatory conditions, aging, and disorders linked to hair growth [1]. However, despite these beneficial effects, the therapeutic potential of LED irradiation remains controversial due to the divergence of protocols used [2,3].

While red light (630–700 nm) is able to reach the dermis, blue light (400–470 nm) has a lower potential for penetration and has been found to be useful for skin conditions in the epidermal layer [2]. Mechanistic studies have shown that LEDs at 625 nm had the potential to treat skin disorders via reactive oxygen species (ROS) and/or inflammatory signaling through the SPHK1/NF-κB pathway [4]. LEDs at 655 nm have promoted human hair growth by activating Wnt/β-catenin signaling [5], and LEDs at 660 nm have reversed collagen downregulation and matrix metalloproteinase-1 upregulation in human skin [6]. LEDs at 464 nm have induced ROS production and the activation of mitogen-activated protein kinase (MAPK), p38, and NF-κB in retinal pigment epithelial (RPE) cells [7]. The blue light treatment of RPE cells increased the expression of Bax, cleaved caspase-3, FasL, and FADD while inhibiting Bcl-2 and Bcl-xL accumulation and the Bcl-2/Bax association [8]. Although the broad range of visible light (400–700 nm, blue, green, and red) could induce ROS generation that has been linked to various cellular mechanisms [7,9,10,11], the individual effects of cell viability in specific light ranges is not yet fully understood.

Except for the optic nerve cells in the eye, the skin is the most extensive tissue that receives light, and light acts upon skin tissue. Therefore, it is noteworthy to examine the effect of LED light on the skin and investigate the specific mechanisms. Recently accumulated evidence has suggested that while red LED light increased cell proliferation, blue LED light decreased cell viability in various cell types via altered mitochondrial functions including aberrant ROS formation [7,8,9,10,11,12].

Focal adhesion kinase (FAK) is a protein tyrosine kinase that is activated via integrins and growth factor receptors [13]. The activation of FAK plays a critical role in cellular migration and proliferation as well as in tumor metastasis and angiogenesis [14,15]. In addition, FAK plays a major role in inflammatory cytokine signaling [16,17]. Interestingly, ROS have been linked to the activation of several protein tyrosine kinases, such as FAK, proline-rich tyrosine kinase 2, and Src [18,19,20,21].

In this study, we first showed that LED light may be a new stimulus for FAK activation via ROS production that regulates various intracellular signal changes including the inhibitory-κB Kinase α (IKKα), and MAPK pathway. Furthermore, we found that red LED light elicited a distinct pattern of FAK activation and downstream signaling, as compared to blue LED light in HaCaT cells. These results suggested that red LED light could be beneficial to cell survival through the differential activations of FAK, IKKα, c-jun N-terminal kinase (JNK), and MAPK signaling.

## 2. Materials and Methods

### 2.1. Cells and Reagents

Human skin keratinocytes, HaCaT cells, were obtained from the American Type Culture Collection (USA) and maintained Dulbecco’s modified essential medium containing 10% fetal bovine (Thermo Fisher Scientific, Waltham, MA, USA), 1 mM sodium pyruvate, 0.1 mM nonessential amino acids, 100 units/mL penicillin, and 100 μg/mL streptomycin. Diphenyliodonium (DPI), N-acetyl-L-cysteine (NAC), and z-DEVD-fmk were purchased from Sigma-Aldrich. FAK inhibitor (FAKi; PF-562,271) was obtained from MedKoo (Chapel Hill, NC, USA). Orange CM-H_2_TMRos and 2′,7′-dichlorodihydrofluorescein diacetate (H_2_DCFDA) and MitoTracker Orange CM-H2TMRos were purchased from Invitrogen (Carlsbad, CA, USA). Antibodies against FAK (Millipore, Burlington, MA, USA), pY397 FAK (Invitrogen), ERK, p-ERK, p-JNK, p-p38, p-IKKα, cleaved caspase-3, PARP (Cell Signaling, Danvers, MA, USA), and GAPDH (Millipore) antibody were obtained.

### 2.2. Cell Viability Assay

Cell viability assay was performed using the thiazolyl blue terazolium bromide (MTT) assay. HaCaT cells were cultured in 96-well plates (dark-sided, clear-bottomed 96-well microplate, Thermo Fisher Scientific, Rochester, NY, USA) at a density of 1 × 10^4^ cells/well and pretreated with or without NAC (10 mM) or DPI (10 μM) for 1 h before cells were exposed to 2500–20,000 lux of red, green, or blue LED light for 1 h. After 24 h, the cells were then incubated with MTT (0.25 mg/mL) at 37 °C in a CO_2_ incubator for 4 h. MTT formazan products were dissolved in dimethyl sulfoxide, and the absorbance was measured at 570 nm using a microplate reader (Bio-Tek Instruments Inc., Santa Clara, CA, USA).

### 2.3. ROS Measurement

HaCaT cells were cultured in 96-well plates (dark-sided, clear-bottomed) at a density of 1 × 10^4^ cells/well and pretreated with or without NAC (10 mM) or DPI (10 μM) for 1 h before cells were exposed to 2500–20,000 lux of red, green, or blue LED light for 1 h. HaCaT cells were then stained for 30 min at 37 °C with 10 µM H_2_DCFDA or 100 nM MitoTracker Orange CM-H2TMRos (Mito-Orange). After staining, the cells were washed with phosphate-buffered saline and analyzed using a fluorescence microplate reader or visualized fluorescence microscopy.

### 2.4. LED Light Exposure

Direct sunlight can reach a light intensity of up to 100,000 lux and up to 25,000 lux in full daylight. Light intensities indoors are considerably lower, and standard office lighting is typically 500 lux or lower. During the day, light levels depend on the presence of clouds and haze, and they may vary minute to minute due to cloud cover, atmospheric turbidity, etc. Therefore, we simulated an overcast day (2500 lux) and full daylight (20,000 lux) for 1 h (Supplemental Table S1).

LEDs (red, 620–630 nm, model GT-P25R1, 10 w; green, 515–525 nm, 10 w, GT-P25G6; blue, 460–470 nm, 9 w, GT-P25WB) were purchased from Shenzhen Getian Opto-Electronics Co., Ltd. (Shenzhen, Guangdong, China) and were attached to a fan and a heat sink to reduce the heat transfer to samples. The HaCaT cells were seeded into a 96-well (for MTT assays or ROS measurement) or 6-well cell-culture plate (for Western blotting or adhesion assays). After 24 h, the cells were exposed to red (627 nm), green (525 nm), or blue (450 nm) LED light at a unified illuminance of 2500–20,000 lux for 1 h in an incubator equipped with an LED box. The light illuminance was measured and adjusted using a lux meter (Hioki 3423 Lux HiTester; Hioki E. E. Corporation, Nagano, Japan) on the sample surface. A plate of cells was also incubated in a dark-maintained incubator, as a control. Control and light-irradiated cells were obtained from the same stock to avoid any pre-existing bias.

### 2.5. Immunoblotting

For LED-dose experiments, HaCaT cells in 6-well plates were exposed to red, green, or blue LED lights at 2500–20,000 lux for 10, 30, and 60 min intervals and then immediately lysed with a radio-immunoprecipitation assay buffer. To observe the cellular effects of ROS in cellular signaling, HaCaT cells in 6-well plates were pretreated with and without NAC (10 mM), DPI (10 μM), PF-271 (1 μM), or DEVD-fmk (200 μM) for 1 h before being exposed to LED lights (20,000 lux) for 10 min and then lysed for the indicated times. Clarified lysates were run on 4%–12% NuPAGE Tris-Bis gels (Invitrogen). Protein was transferred to polyvinylidene fluoride membranes, blocked with 3% bovine serum albumin, and incubated overnight with primary AT antibodies at 4 °C. Membranes were washed with Tris-buffered saline with 0.1% Tween^®^ 20 detergent and incubated with HRP-conjugated secondary antibodies, and then the proteins were visualized using Enhanced chemiluminescence on the ChemiDoc MP Imaging System (Bio-Rad, Hercules, CA, USA).

### 2.6. Cell Detachment Assay

HaCaT cells in 6-well plates were exposed to 20,000 lux LED lights (red, green, or blue) for 1 h and then treated with or without z-DEVD-fmk (200 μM) for 24 h. Floating (detached cells in the supernatant) and adherent cells (obtained by trypsinization) were harvested separately after LED lights exposure for 24 h. Cell counts were determined using a hemocytometer and trypan blue exclusion method. All viable (unstained) and dead (stained) cells were counted, which was followed by the determination of the percentage of cell adhesion and detachment using the following equation: % of adherent cells = (number of adherent cells/total number of cells) × 100%; % of detached cells = (number of floating cells/total number of cells) × 100%.

### 2.7. Statistical Analysis

Statistical significance between experimental groups was determined using the Student’s *t*-test or two-way analysis of variance (ANOVA) with the Sidak multiple comparisons test (Prism software, v7.0d; GraphPad Software, La Jolla, CA, USA).

## 3. Results

### 3.1. LED Light Differentially Regulated Cell Viability

To investigate the potentially differential effects of LED lights (red, green, and blue), we used HaCaT cells in vitro. After LED light exposure, the viable cells were measured via MTT assay.

**Figure 1:**
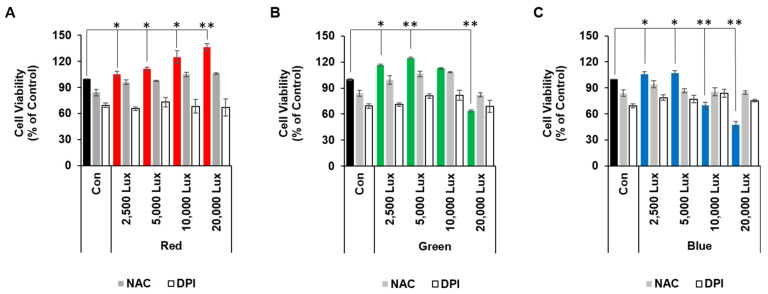
Differential effects of red, green, and blue LED light exposure on the viability of HaCaT cells. HaCaT cells cultured in a 96-well plate were pretreated with or without NAC (10 mM) or DPI (10 μM) for 1 h prior to exposure with red (**A**), green (**B**), or blue (**C**) LED lights for 1 h. After 24 h, cell viability was quantified by MTT assay (*n* = 3, ±SD). * *p* < 0.01 and ** *p* < 0.001 vs. control.

Red LED light (at 2500–20,000 lux) and green or blue LED light at only lower light intensities (at 2500 and 5000 lux, respectively) gradually increased cell viability, as compared to controls. However, a higher intensity of green or blue LED light reduced cell viability (green at 20,000 lux or blue at 10,000 and 20,000 lux), as compared to controls (Figure 1). As LED light exposure in various types of cells can cause ROS generation, we next examined the effects of LED-mediated ROS production on cell viability. To reduce cellular ROS levels, we used NAC (an ROS scavenger) and DPI (a NOX inhibitor), which can reduce cellular and mitochondrial ROS production [21]. Both NAC and DPI treatments slightly lowered cell viability in controls (Figure 1).

While red LED at all ranges as well as green and blue LED exposure at 2500 and 5000 lux increased cell viability (Figure 1), green LED exposure at 20,000 lux and blue LED exposure at 10,000 and 20,000 lux decreased cell viability. However, the NAC and DPI treatments improved the decreased cell viability during green LED exposure at 20,000 lux and blue LED exposure at 10,000 and 20,000 lux (Figure 1). Therefore, these results indicated that depending on the intensity and wavelength, LED lights may affect cell viability either positively or negatively as a result of cellular ROS formation.

### 3.2. LED Light Stimulated Cellular ROS Formation

**Figure 2:**
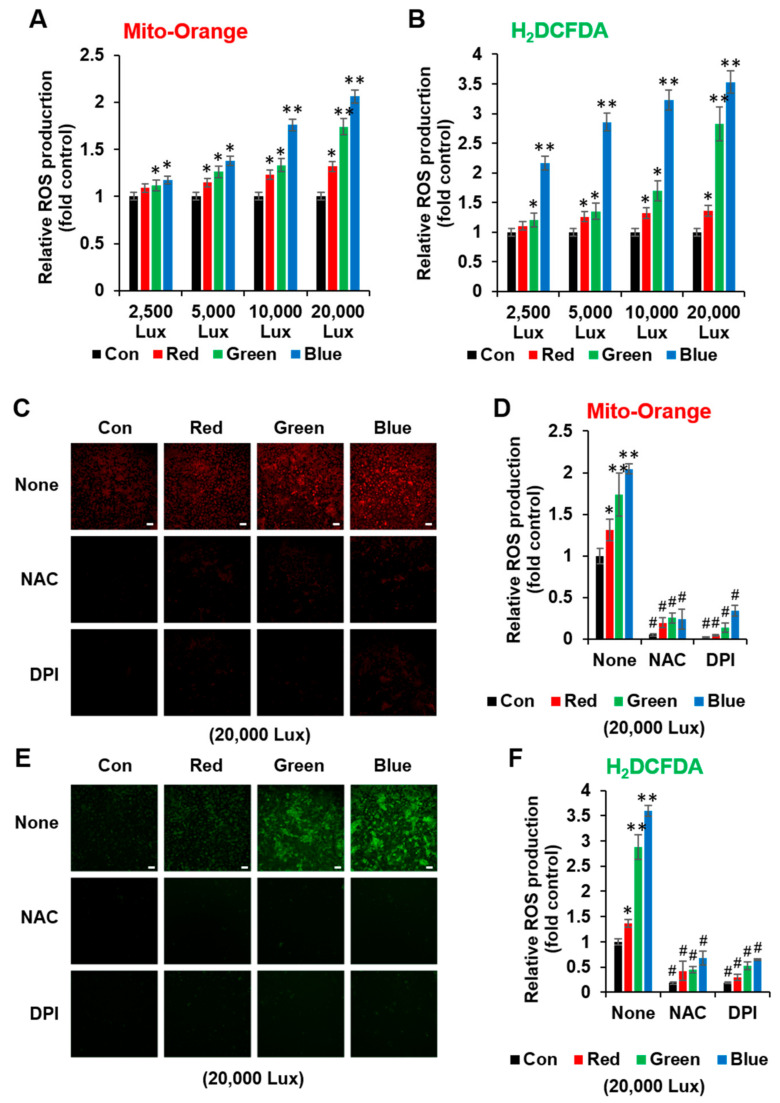
Red, green, and blue LED light exposure produced different levels of ROS. HaCaT cells cultured in a 96-well plate were exposed to indicated LED lights for 1 h and then stained by Mito-Orange (**A**,**C**,**D**) or H_2_DCFDA (**B**,**E**,**F**). HaCaT cells were pretreated with NAC (10 mM) or DPI (10 μM) for 1 h before cells were exposed to LED lights (**C**–**F**). ROS production was measured by using a fluorescence plate reader (**A**,**B**) or fluorescence microscopy. Scale bar, 100 μm. (**C**,**E**) and plotted as a bar graph (**D**,**F**) to present the fold changes in ROS production in samples exposed to LED lights, relative to unexposed control cells (*n* = 3, ±SD). * *p* < 0.01 and ** *p* < 0.001 vs. control. None vs. NAC or DPI, ^#^ *p* < 0.001.

To test our hypothesis that different levels of ROS may be produced by various LED lights, we measured the cellular ROS formation by using Mito-Orange or H_2_DCFDA under different LED light conditions. While red LED light slightly increased ROS levels with increasing intensity of LED light, exposure to either green or blue LED light led to a sharp increase in ROS levels (Figure 2A,B). In addition, the high levels of ROS generated when HaCaT cells were exposed to 20,000 lux of green or blue LED light were blocked by either the NAC or DPI treatment (Figure 2C–F). Therefore, these results indicated that low levels of red LED-induced ROS may enhance cell viability, but high levels of green and blue LED-induced ROS may induce cellular stress or death. Interestingly, the ROS values obtained by Mito-Orange (mitochondrial ROS marker) was lower than that from DCF (cellular ROS marker), despite similar trends. This difference may have been due to cellular ROS formation by LED light that could be generated not only from the mitochondria but also via NADPH oxidase-dependent mechanisms.

### 3.3. LED LightStimulated FAK and MAPKs Activation

**Figure 3:**
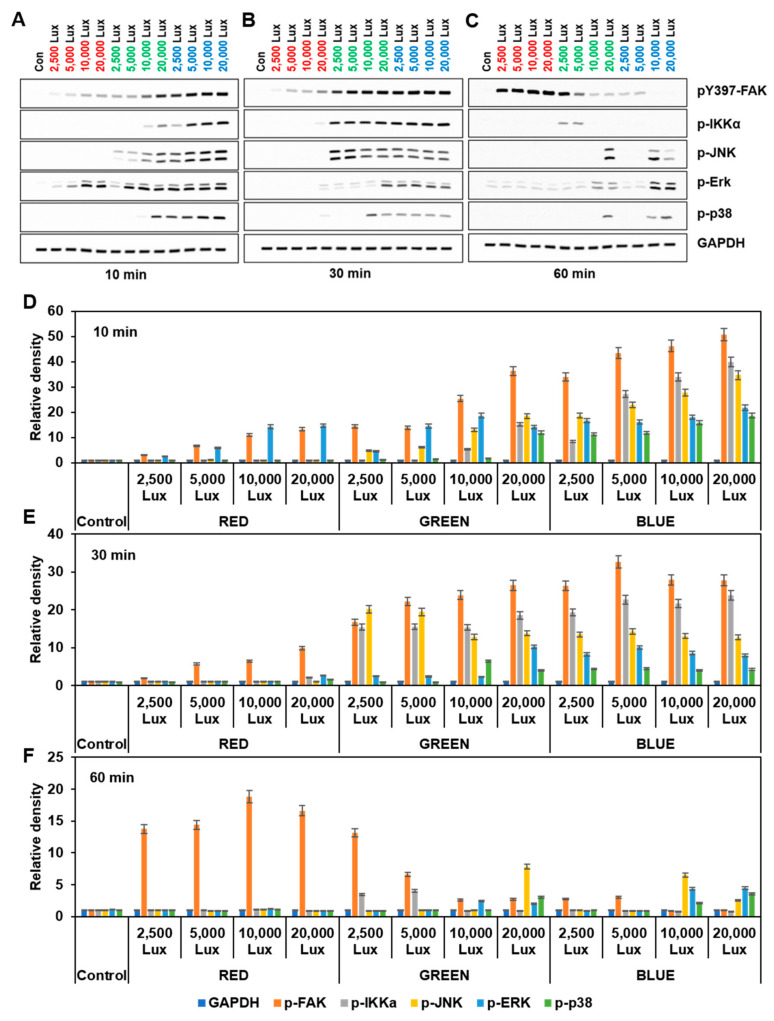
Effect of red, green, and blue LED lights on FAK and MAPK activation. HaCaT cells in a 6-well plate were exposed to the indicated LED lights for the indicated times. (**A**–**C**) Shown are immunoblots of pY397 FAK, p-IKKα, p-JNK, p-ERK, and p-p38, as well as GAPDH as a loading control. (**D**–**F**) Fold changes of pY397 FAK, p-IKKα, p-JNK, p-ERK, and p-p38 were calculated (*n* = 3, ±SD).

We recently reported that tyrosine kinases including FAK were activated by PMA-induced ROS in HT-29 cells, and many studies have shown crosstalk between ROS and FAK [22]. Therefore, we evaluated the effect of LED lights on FAK activity in HaCaT keratinocytes. FAK activity (monitored by pY397 FAK autophosphorylation) was evaluated by LED light intensity during a time course, and the cell lysates were analyzed by Western blotting. The levels of FAK pY397 were rapidly increased by a 10 min exposure of all wavelength ranges of LED lights and were dependent on LED intensity (Figure 3). FAK activity rapidly and robustly increased in the order of blue, green, and red LED lights.

Recent studies have suggested that red LED light stimulated ERK activation and promoted cell proliferation, while blue LED light induced the inflammation pathway in various cell types [7,8,10,11]. Upon red LED light exposure, phosphorylation of ERK was rapidly increased at 10 min and then decreased after 30 min. In green or blue LED-exposed cells, phosphorylation of IKKα, JNK, p38, and ERK was observed at 10 min and was sustained during intervals of 30 and 60 min (Figure 3). These results suggested that LED-light-induced cellular signaling appeared to differ depending on the wavelength of the LED lights.

### 3.4. FAK as an Upstream of MAPK in LED-Induced Signaling

**Figure 4:**
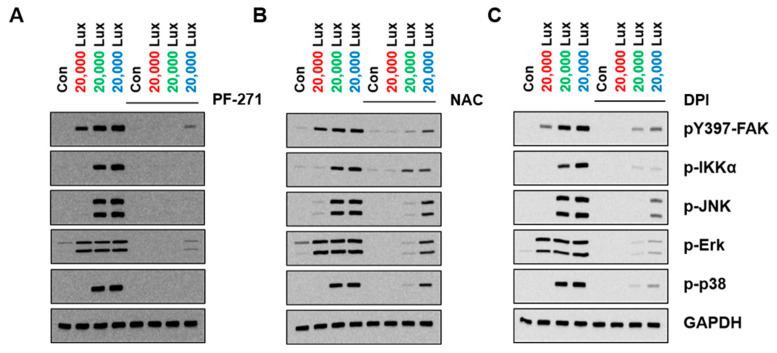
FAK inhibition or ROS scavengers reduced LED light-induced MAPKs activation. HaCaT cells in a 6-well plate were pretreated with FAK inhibitor (**A**) NAC (10 mM) (**B**) or DPI (10 μM) (**C**) for 1 h and then were exposed to LED lights for 10 min. Shown are immunoblots of pY397 FAK, p-IKKα, p-JNK, p-ERK, and p-p38, as well as GAPDH as loading control.

Since MAPK signaling has been critically associated with FAK in cellular physiological functions, we investigated whether FAK regulated LED-mediated MAPK activation via ROS. Treatment with an FAK inhibitor (PF271) blocked the phosphorylation of MAPKs (Figure 4A, Supplemental Figure S1). The NAC and DPI treatments reduced LED-induced activation of MAPKs by decreasing FAK activity (Figure 4B,C, Supplemental Figures S2 and S3). Together, these data suggested that LED-induced ROS activated FAK and regulated downstream MAPK signaling in HaCaT cells.

### 3.5. FAK Activation Was Induced Differently Depending on the Wavelength of the LED Light

We observed that red LED light increased cell viability, but green and blue LED lights decreased it. As the initial activations of FAK and ERK were observed in all the ranges of LED wavelengths and to better explain the differential effects on cell viability, we checked whether the long-term activation of FAK and ERK would differ upon exposure to red, green, and blue LED lights.

**Figure 5:**
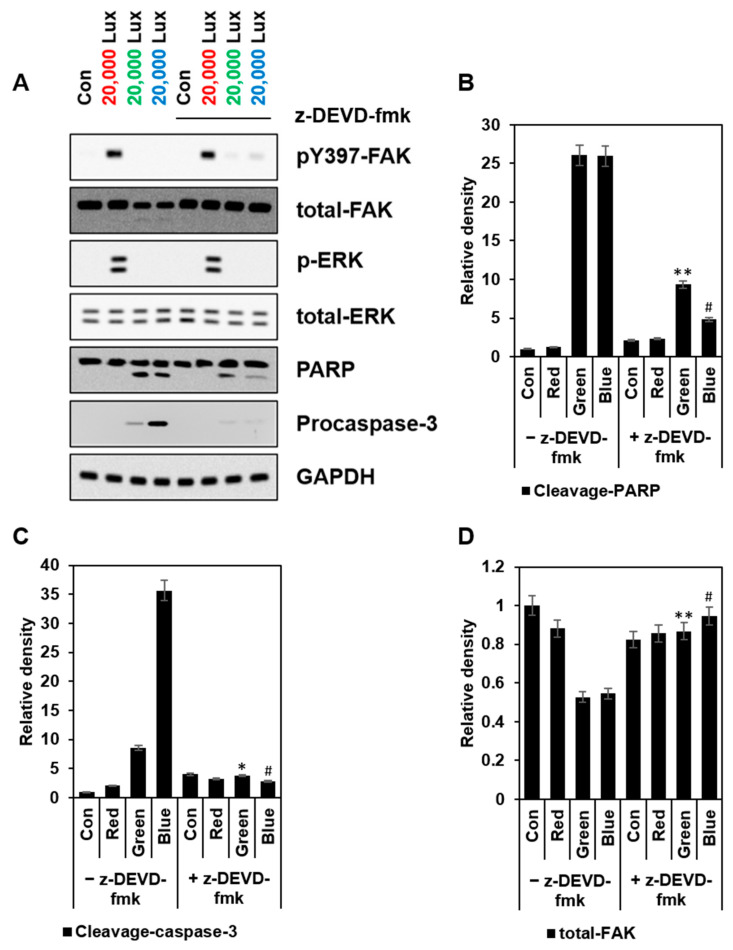
High intensity of green and blue LED light induced FAK inactivation and caspase-3 activation. HaCaT cells in a 6-well plate were exposed to the indicated LED lights (red, green, or blue) for 1 h and then treated with or without z-DEVD-fmk (200 μM) for 24 h. (**A**) Shown are immunoblots of pY397 FAK, total-FAK, p-ERK, total-ERK, PARP, and active caspase-3 (cleaved), as well as GAPDH as loading control. (**B**–**D**) Fold changes of pY397 FAK, p-IKKα, p-JNK, p-ERK, and p-p38 were calculated (*n* = 3, ±SD). LED (Green)—z-DEVD-fmk vs. LED (Green) + z-DEVD-fmk, * *p* < 0.01 and ** *p* < 0.001. LED (Blue)—z-DEVD-fmk vs. LED (Blue) + z-DEVD-fmk, ^#^ *p* < 0.001.

When we compared collected lysates from each group after 24 h of 1 h LED exposure, the red LED group sustained strong activation of FAK and ERK, but the green and blue LED group exhibited no activity or very minimal activation of both kinases. In addition, green and blue LED lights increased cleaved PARP and caspase-3, and the treatment of z-DEVD-fmk (a caspase-3 inhibitor) partially reduced caspase-3 cleavage (Figure 5).

**Figure 6:**
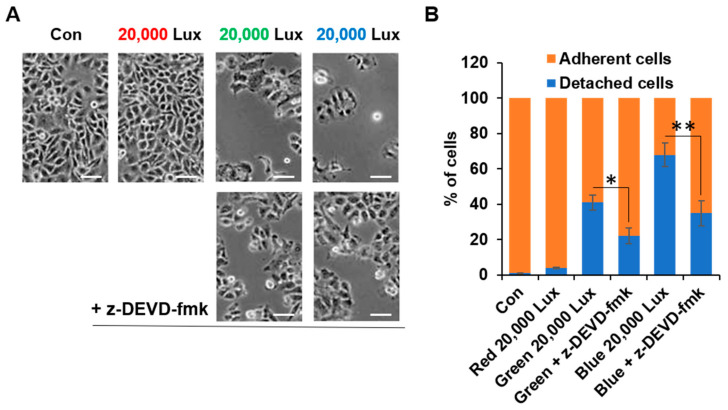
Green and blue LED light exposure increased cell detachment. HaCaT cells in a 6-well plate were exposed to the indicated LED lights for 1 h and then treated with or without z-DEVD-fmk (200 μM) for 24 h (**A**,**B**). Representative phase-contrast images are shown (**A**) and percentage of cell adhesion and detachment relative to control (**B**) was quantified (*n* = 3, ±SD). Scale bar, 100 μm. * *p* < 0.01 and ** *p* < 0.001 vs. control.

Since sustained FAK activation may contribute to enhanced cell viability, we next investigated cell adhesion abilities among the different LED-exposed groups. When exposed to green and blue LEDs, the adhesion ability of cells decreased, and the number of detached cells increased. However, these negative effects were significantly reduced by z-DEVD-fmk treatment (Figure 6). Therefore, our results indicated that exposure to high intensities of green and blue LED lights led to impairment of cell adhesion due to inactivation of FAK.

Altogether, our results suggested that red LED lights induced mitogenic effects via a low-level ROS-FAK-ERK axis, while green and blue LED lights induced cytotoxic effects via apoptosis signaling by generating high levels of ROS.

## 4. Discussion

In this study, we observed that LEDs induced ROS formation and activated FAK in HaCaT cells. Since FAK is a major upstream signal molecule and is involved in various pathophysiological mechanisms, our findings could expand PDT studies with FAK-associated mechanisms.

The homeostasis of cellular ROS formation has been shown to be important for cell viability [23], and typically, low-level ROS is essential for cell homeostasis while high levels of ROS have caused negative effects with a reduction in ATP synthesis [24]. Concomitantly, our results showed that red LED light induced lower levels of ROS formation and increased cell viability, while green and red LED lights induced higher levels of ROS formation and decreased cell viability (Figure 1 and Figure 2). Therefore, red LED light may be beneficial to the homeostasis of HaCaT cells, while green and blue LED lights may have cytotoxic effects.

Since ROS formation via a light source has been identified in mitochondria [25], NAC effects are expected in LED-exposed cells, but DPI effects should be considered. Although DPI can inhibit mitochondrial ROS generation, its major role is acting as a NOXs inhibitor. HaCaT cells have Nox1,2,5 that are regulated by various stimulus [26,27]. Especially, blue LED light-induced ROS formation mitigated via DCF was higher than via using Mito-orange, and these effects were blocked by DPI (Figure 2). This may suggest that blue LED light induces mitochondrial ROS, in addition to another ROS source, NOXs. Therefore, further studies could examine the activation of NOXs- and NOX-associated molecules (e.g., NoxO, NoxA, and Rac1) via LED lights.

As FAK is an upstream signal molecule, it is regulated by various downstream molecules including IKKα, ERK, JNK, and p38 as well as direct effectors [28,29]. It is activated by various receptors with extracellular molecules, and ROS has been known to activate FAK via the oxidation and the inhibition of specific phosphatase for FAK and oxidation Src [19,20]. We showed that FAK was activated by various LED wavelengths and intensities, but there were differences in the timing of the activation of FAK. Red LED light slowly increased FAK phosphorylation, while green and blue LED lights strongly and transiently stimulated FAK phosphorylation (Figure 3). These results suggested that FAK activation may be dependent on the amount and the timing of the generated ROS.

The activity of ERK was shown to be similar to that of FAK. Phosphorylation of IKKα, JNK, and p38 (i.e., markers of inflammation and cellular stress) was observed by green and blue LED lights, while it was not detected after exposure to red LED light (Figure 3). However, these effects were inhibited by an FAK inhibitor (PF-271) or an ROS inhibitor (i.e., NAC and DPI) (Figure 4). These results indicate that FAK had multiple functions in downstream signaling, and LED lights could regulate FAK-associated signaling events via ROS in various ways.

Many studies have suggested that FAK had an anti-apoptotic role in anchorage-dependent cells [30], and this was mediated by death-receptor-related signaling pathways [31]. Therefore, it was presumed that the early FAK–JNK and FAK–p38 pathways activated by green or blue LED illumination did not induce apoptosis, but rather induced cell migration and inflammatory responses by FAK activity. Therefore, apoptosis by green or blue LED illumination was triggered by excessive intracellular ROS. However, since the movement of skin cells and the inflammatory response are important for various skin physiology and diseases, it could also be useful to study the wavelengths and intensities of LED lights that induce cell migration and inflammation without apoptosis.

**Figure 7:**
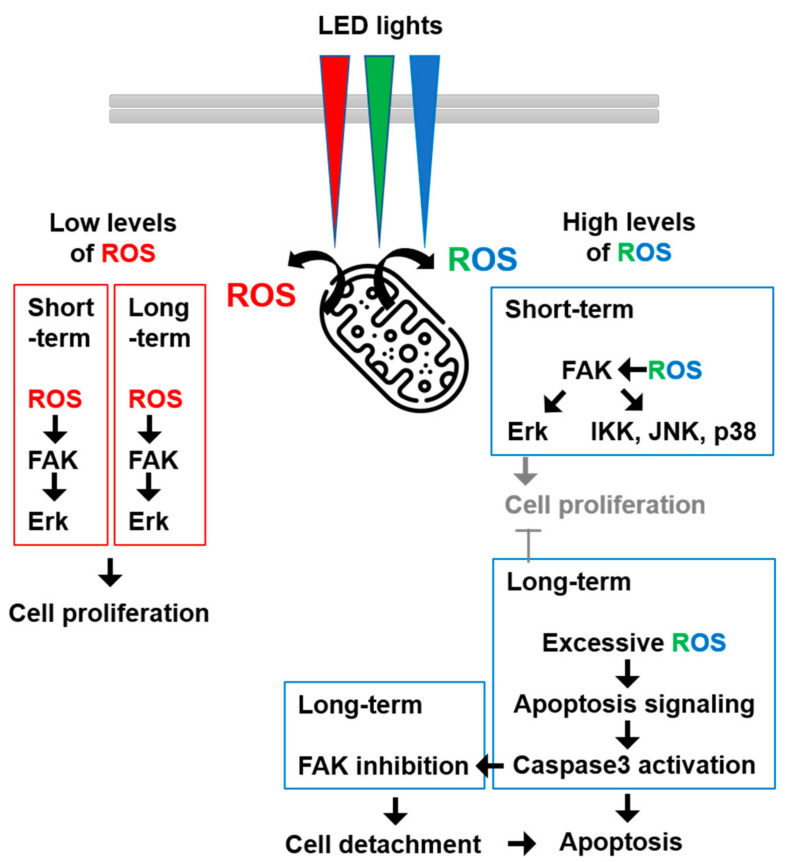
LED light exposure modulated FAK-related signaling events differently via ROS in HaCaT cells. Red LED light induced mitogenic effects via a low-level ROS-FAK-ERK axis, while green and blue LED lights induced cytotoxic effects via apoptosis signaling by generating high levels of ROS.

FAK cleavage is mediated by caspases-3, and FAK contributes to the morphological changes that have been observed in apoptotic suspension and adherent cells [32]. Inhibition of FAK has induced interference in cell proliferation and adhesion, eventually leading to anoikis [33], as seen in our results (Figure 5 and Figure 6). In the results of the Western blotting (Figure 5A), not only was the FAK activity inhibited by the green and blue LED lights, but also the amount of total FAK was decreased. When caspase-3 activity was blocked by z-DEVD-fmk treatment, the activity and amount of FAK were restored to some extent. Therefore, these results indicated that excessive ROS caused by green or blue LED light activated caspase-3 and inhibited the functions of FAK, such as ERK activation. This result was also connected with the cell detachment data in Figure 6. Taken together, our results suggested that LED lights were new stimuli for FAK activation and could control FAK-involved cellular signaling mechanism in HaCaT cells (Figure 7).

## Data Availability

The data presented in this study are available in this article and Supplementary Materials.

## Supplementary Materials

The following supporting information can be downloaded at: https://www.mdpi.com/1467-3045/44/3/82/s1, Table S1. Illuminance provided under various conditions; Figure S1. FAK inhibition reduced LED-induced MAPKs activation; Figure S2. ROS scavenger (NAC) reduced LED-induced MAPKs activation; Figure S3. ROS scavenger (DPI) reduced LED light-induced MAPKs activation.

## Abbreviations

DPI: Diphenyliodonium; ERK: Extracellular-signal-regulated protein kinase; FAK: Focal adhesion kinase; IKKα: Inhibitory-κB Kinase α; JNK: c-jun N-terminal kinase; LED: Light-emitting diode; MAPK: Mitogen-activated protein; NAC: N-acetyl-L-cysteine; PDT: photodynamic therapy; ROS: reactive oxygen species.
