# Supplementary material for: LED Light-Induced ROS Differentially Regulates Focal Adhesion Kinase Activity in HaCaT Cell Viability
Source: Curr Issues Mol Biol. 2022 Mar 4;44(3):0. doi: 10.3390/cimb44030082 (PMC8947587; doi:10.3390/cimb44030082)
Supplement: Supplementary file 1 [file cimb-44-00082-s001.zip › Supplemental Information (KJS).pdf]

Supplemental Information

Title: LED lights-induced ROS differentially regulates focal adhesion kinase activity in HaCaT cell viability

Author's name:

Jun-Sub Kim[1], and Ssang-Taek Steve Lim[2]

Affiliation:

1. Department of Biotechnology, Korea National Transportation University, Chungbuk, Korea

2. Department of Biochemistry and Molecular Biology, University of South Alabama, College of Medicine, Mobile, AL 36688

Correspondence should be addressed to Jun-Sub Kim; junskim@ut.ac.kr and Ssang-Taek Steve Lim; stlim@southalabama.edu

Running Title: LED lights regulate FAK activity

Supplemental Table S1. Illuminance provided under various conditions (from Wikipedia, Lux)

**Illuminance (lux)**	**Surfaces Illuminated by**
0.0001	Moonless, overcast night sky (starlight)
0.002	Moonless clear night sky with airglow
0.05–0.3	Full moon on a clear night
3.4	Dark limit of civil twilight under a clear sky
20–50	Public areas with dark surroundings
50	Family living room lights
80	Office building hallway/toilet lighting
100	Very dark overcast day
150	Train station platforms
320–500	Office lighting
400	Sunrise or sunset on a clear day.
1000	Overcast day; typical TV studio lighting
10,000–25,000	Full daylight (not direct sun)
32,000–100,000	Direct sunlight

Supplemental Figure legends

Supplemental Figure S1. FAK inhibition reduces LED light-induced MAPKs activation. HaCaT cells in a 6-well plate were pretreated with FAK inhibitor (A) for 1 h and then were exposed to LED lights for 10 min. Shown are immunoblots of pY397 FAK, p-IKKα, p-JNK, p-ERK, p-p38 and GAPDH as loading control. (a-f) Fold change of pY397 FAK, p-IKKα, p-JNK, p-ERK, and p-p38 was calculated (n=3, ±SD). ** $p < 0.001$ vs. control. LED (Red, Green, or Blue) vs. LED (Red, Green, or Blue) + FAKi, NAC, or DPI, $^{\#}$ $p < 0.001$

Supplemental Figure S2. ROS scavenger (NAC) reduces LED light-induced MAPKs activation. HaCaT cells in a 6-well plate were pretreated with NAC (10 mM) (A) for 1 h and then were exposed to LED lights for 10 min. Shown are immunoblots of pY397 FAK, p-IKKα, p-JNK, p-ERK, p-p38 and GAPDH as loading control. (a-f) Fold change of pY397 FAK, p-IKKα, p-JNK, p-ERK, and p-p38 was calculated (n=3, ±SD). ** $p < 0.001$ vs. control. LED (Red, Green, or Blue) vs. LED (Red, Green, or Blue) + FAKi, NAC, or DPI, $^{\#}$ $p < 0.001$

Supplemental Figure S3. ROS scavenger (DPI) reduces LED light-induced MAPKs activation. HaCaT cells in a 6-well plate were pretreated with DPI (10 μM) (A) for 1 h and then were exposed to LED lights for 10 min. Shown are immunoblots of pY397 FAK, p-IKKα, p-JNK, p-ERK, p-p38 and GAPDH as loading control. (a-f) Fold change of pY397 FAK, p-IKKα, p-JNK, p-ERK, and p-p38 was calculated (n=3, ±SD). ** $p < 0.001$ vs. control. LED (Red, Green, or Blue) vs. LED (Red, Green, or Blue) + FAKi, NAC, or DPI, $^{\#}$ $p < 0.001$
