# Supplementary figures and images for: LED Light-Induced ROS Differentially Regulates Focal Adhesion Kinase Activity in HaCaT Cell Viability
Source: Curr Issues Mol Biol. 2022 Mar 4;44(3):0. doi: 10.3390/cimb44030082 (PMC8947587; doi:10.3390/cimb44030082)

**A**

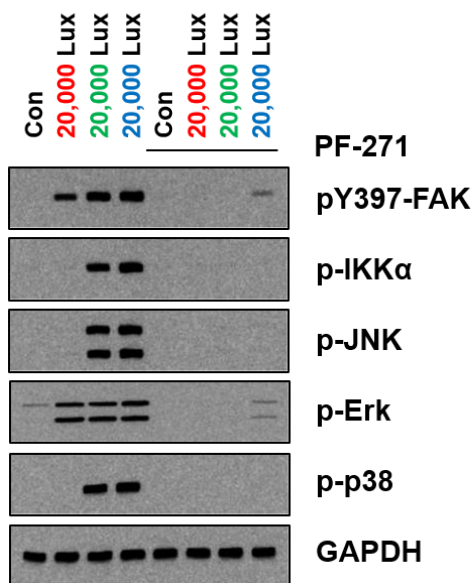

**(a)**

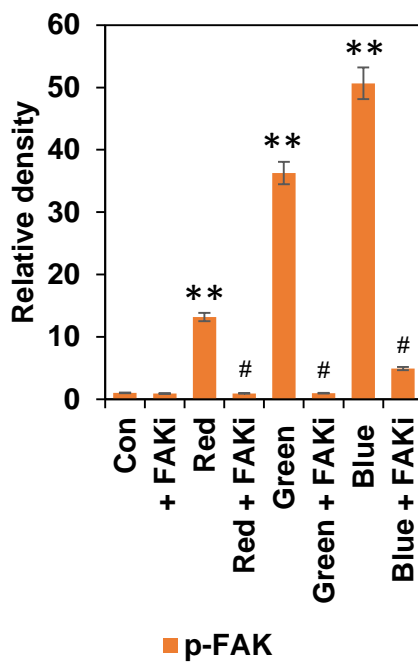

**(b)**

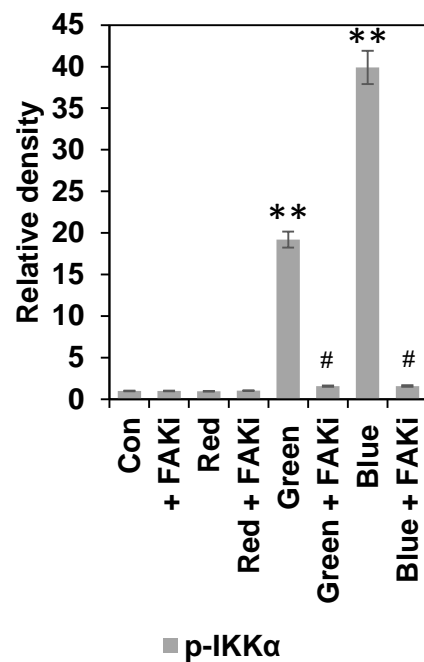

**(c)**

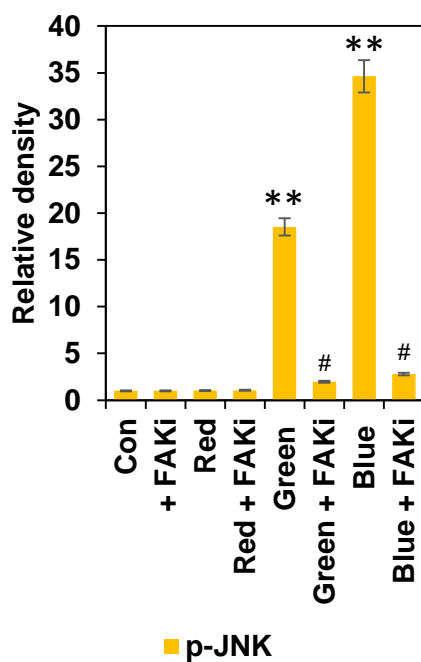

**(d)**

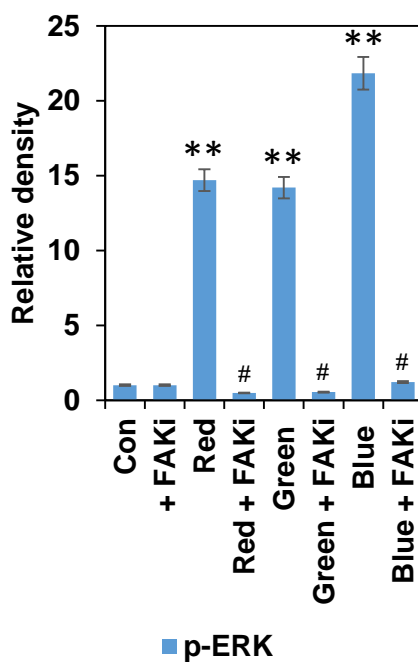

**(e)**

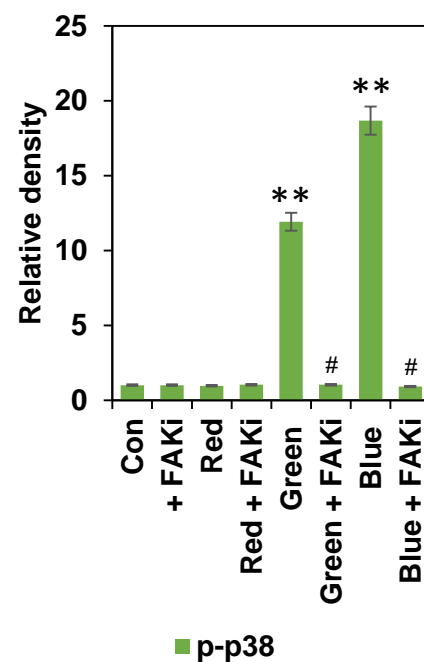

**B**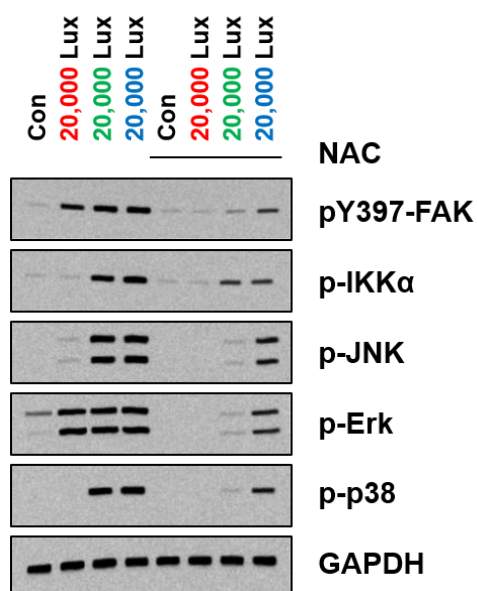**(a)**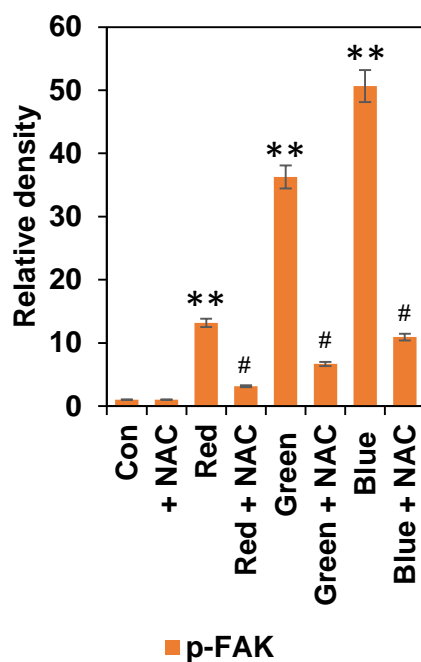**(b)**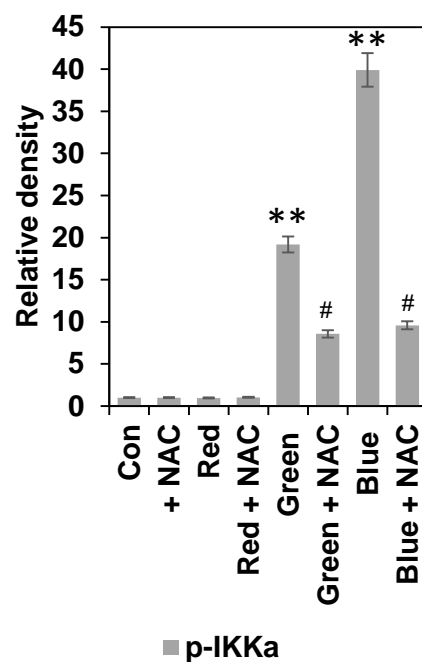**(c)**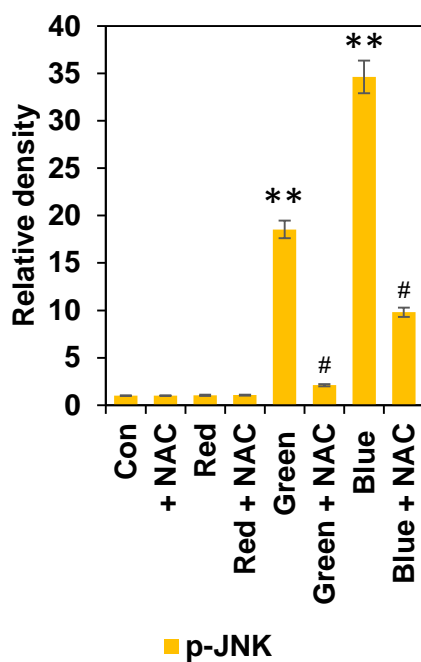**(d)**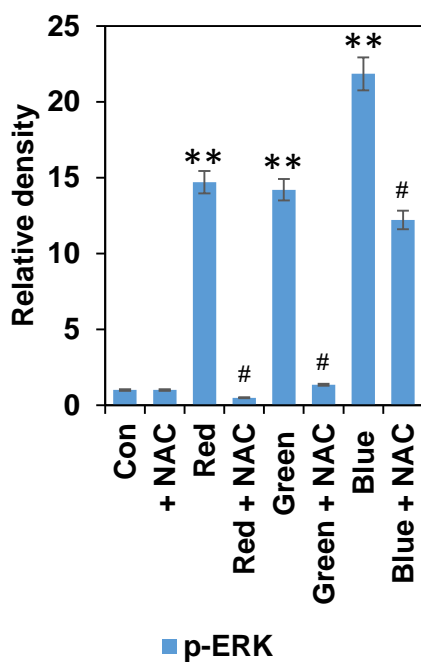**(e)**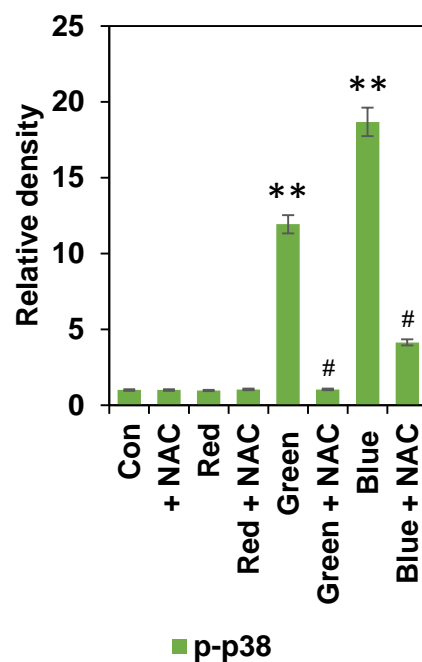

**C**

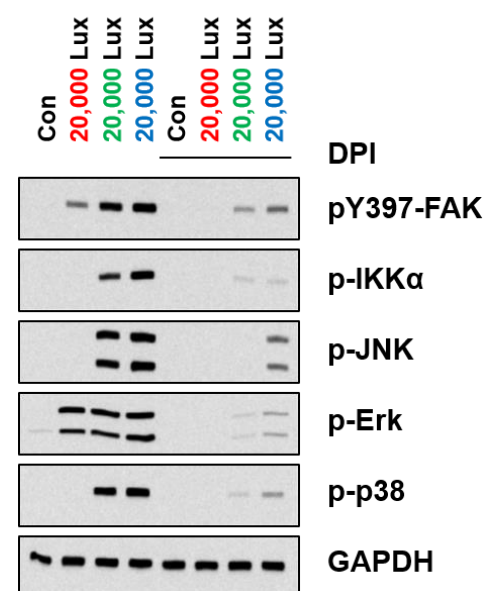

(a)

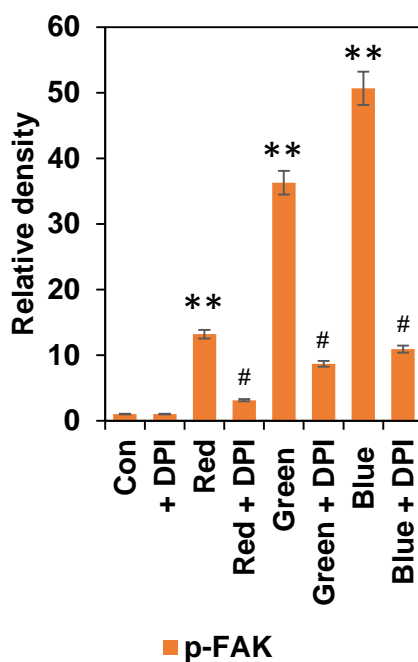

(b)

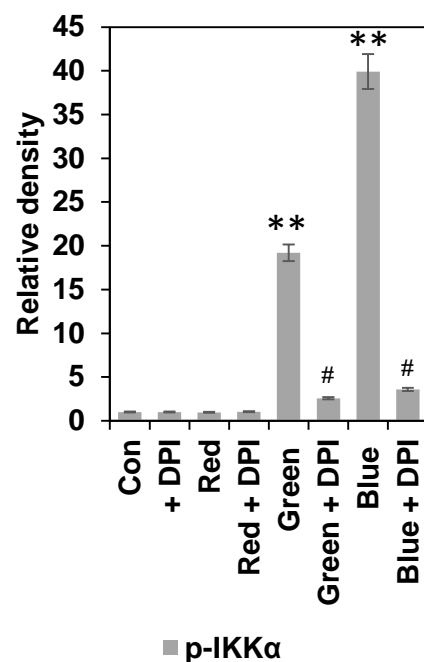

(c)

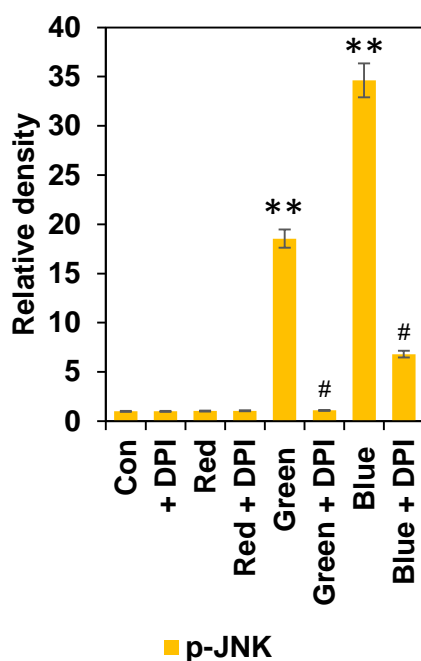

(d)

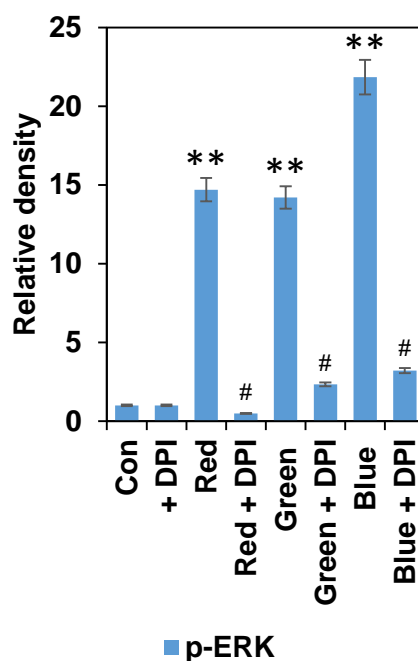

(e)

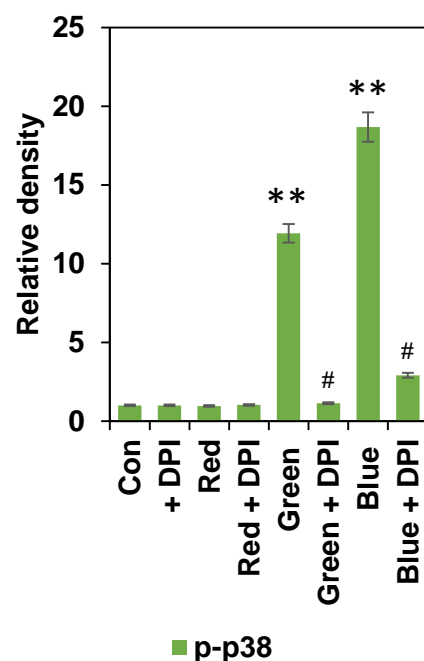

**Supplemental Figure S3**

Supplement: Supplementary file 1 [file cimb-44-00082-s001.zip › Supplemental figures.pdf]
